# The TP53 gene contains a diversity box that makes it more than a tumor suppressor

**DOI:** 10.1038/s41418-026-01681-1

**Published:** 2026-02-13

**Authors:** Arnold J. Levine, Pierre Hainaut

**Affiliations:** 1 Institute for Advanced Study, Princeton, NJ USA; 2 Institute for Advanced Biosciences, Université Grenoble Alpes, La Tronche, France

**Keywords:** Cancer genetics, Cancer genomics

## Abstract

The p53 protein is a transcription factor that is most commonly known and studied as a tumor suppressor preventing cancers. At least 50% of cancers have *TP53* mutations that inactivate p53 functions and many other cancers utilize physiological means to inactivate p53 tumor suppressor functions, pointing to the fundamental role of p53 as a barrier to the expansion of transformed cells. An intriguing characteristic of the *TP53* gene is the presence of multiple regulatory structures that control the diversity of p53 expression within a short region of 1200 bp between exon 2 and exon 5. This “diversity box” encompasses a dense array of regulatory signals that control the selective activation and cell-fate specificity of p53 suppressor functions. These signals include multiple post-translational modification sites and motifs regulating the expression of N-terminally truncated p53 isoforms Δ40p53α and Δ133p53α, all of which contribute to modulate p53 functions and phenotypes. These functions may play a role in early development, DNA repair, wound healing versus cell death, as well as enhanced metabolic and mitochondrial functions, reversal of cellular senescence, cell-type- specific functions, communication with the immune system, and even accelerated aging. Remarkably, the *TP53* diversity box harbors several frequent polymorphisms known to modulate p53 functions, which are in linkage disequilibrium and specify unique haplotypes that differ from one population to another. This suggests that genetic variations in the diversity box may contribute to differences in epidemiological and pathological traits of the same cancer types among individuals from different ancestries.

## Facts

1. The *TP53* gene is a tumor suppressor gene.
2. This gene can express 12 splice variants and isoform proteins.
3. The protein can form monomers, dimers and tetramers.
4. The human *TP53* gene has 14 polymorphisms with known phenotypes.
5. There are 315 different protein combinations that can be formed from this diversity.
6. Most of this diversity resides in 1200 bp of the 20,000 bp *TP53* gene.

## Questions

1. How many different haplotypes of the p53 protein are found in the human population?
2. How do some of the polymorphisms in the *TP53* gene regulate which splice forms are produced?
3. How do mixed tetramers of full length p53 protein dimers and p53 isoforms produce new transcriptional outcomes?
4. Do p53 isomers produce hetero-tetramers with full length p63 or p73 proteins in cancers?
5. What do the *TP53* splice forms and polymorphisms contribute to cancer phenotypes?
6. Do *TP53* polymorphisms that differ among individuals of different ancestries give rise to different cancer phenotypes?

## Introduction

### How much functional Information is contained in the *TP53* Gene?

In 1948, the mathematician/engineer Claude Shannon, who worked at Bell Laboratories in New Jersey, USA, elucidated a set of equations that described and quantified information transfer and its distortion upon transferring that information from one place to another [1]. To identify and quantitate the distortion or uncertainty of the information transfer, he introduced the term p log p (the probability of uncertainty of faithful transmission, times the log of that probability) which it turns out was the equivalent of the entropy of that system, now termed Shannon entropy. Just five years later, in 1953, Watson and Crick published a model of the structure of DNA, based upon the work of R. Franklin, that proposed the mechanisms for both faithful replication of the DNA and the method of storage of information in the sequence of nucleotides of the DNA strands [2]. For the science of biology, the mechanisms of information storage, transfer and translation into functions were recognized for the first time at the molecular level and a part of biology became the study of information transfer and information use. This was accomplished by the deciphering of the genetic code, an alphabet or algorithm that is employed universally by all life forms on earth. By the start of the 21^st^ century, the DNA sequences of *Drosophila melanogaster* and *Caenorhabditis elegans* were elucidated, and *Drosophila* had about 14,000 protein coding genes, whereas *Caenorhabditis* had about 20,000 protein coding genes. So, when the human genome was being sequenced most of the bets about the number of protein coding genes (functional units of information) were about 100,000 such genes or more. However, when the first drafts of the human genome sequences were published it was a surprise to observe only about 25,000 protein coding genes [3, 4]. That first draft of the genome had non-contiguous regions in several chromosomes and repetitive sequences that were not inserted properly into the genome. The now available more complete version of human DNA sequences has about 25,000 protein coding genes and about 10,000 RNA coding genes that are often duplicated with small variations up to 30,000 RNA coding genes commonly used to regulate other genes.

The exploration of one of these human genes, the *TP53* gene, has begun to demonstrate diverse multiple pathways of genes, and their proteins, that turn the functional p53 protein on and off, and then a chemically modified p53 protein making decisions about which signal transduction pathways are taken by a cell, each pathway affecting a different outcome. The p53 protein has 12 isoforms created by splicing of the m-RNA. It also is composed of monomeric, dimeric, and tetrameric forms of the protein that have differential activities, and a surprisingly large number of genetic polymorphisms localized in the introns and exons of 1,200 base pairs including *TP53* introns and exons 2-5 (called the diversity box) out of a gene composed of eleven exons in 20,000 base pairs. These combinatorial mixtures of isoforms, oligomers, and polymorphisms, some with different demonstrated outcomes, create a great deal of functional diversity in one protein coding gene, giving rise to 315 different possible protein combinations. Some of the p53 isoforms may enter tetrameric complexes with any of three closely related transcription factors, p53, p63, or p73. In addition, the p53 protein binds to a large number of additional proteins in cells such as MDM-2, Gas-1, FAK, LATS2 and PTPN14 that inhibit YAP/TAZ of the HIPPO pathway. Most of these combinations, created by a random evolutionary process (mutations, gene duplications) followed by a selection process, may or may not form viable protein functions. Some of these viable protein functions may be indicated by a strong linkage disequilibrium localized across the *TP53* gene in the diversity box, which by preventing viable recombination events, selects for and then limits some combinatorial diversity. This review provides evidence for the functional diversity of the *TP53* gene and explores its consequences.

### The classical functions of the full-length *TP53* gene

**Fig. 1:**
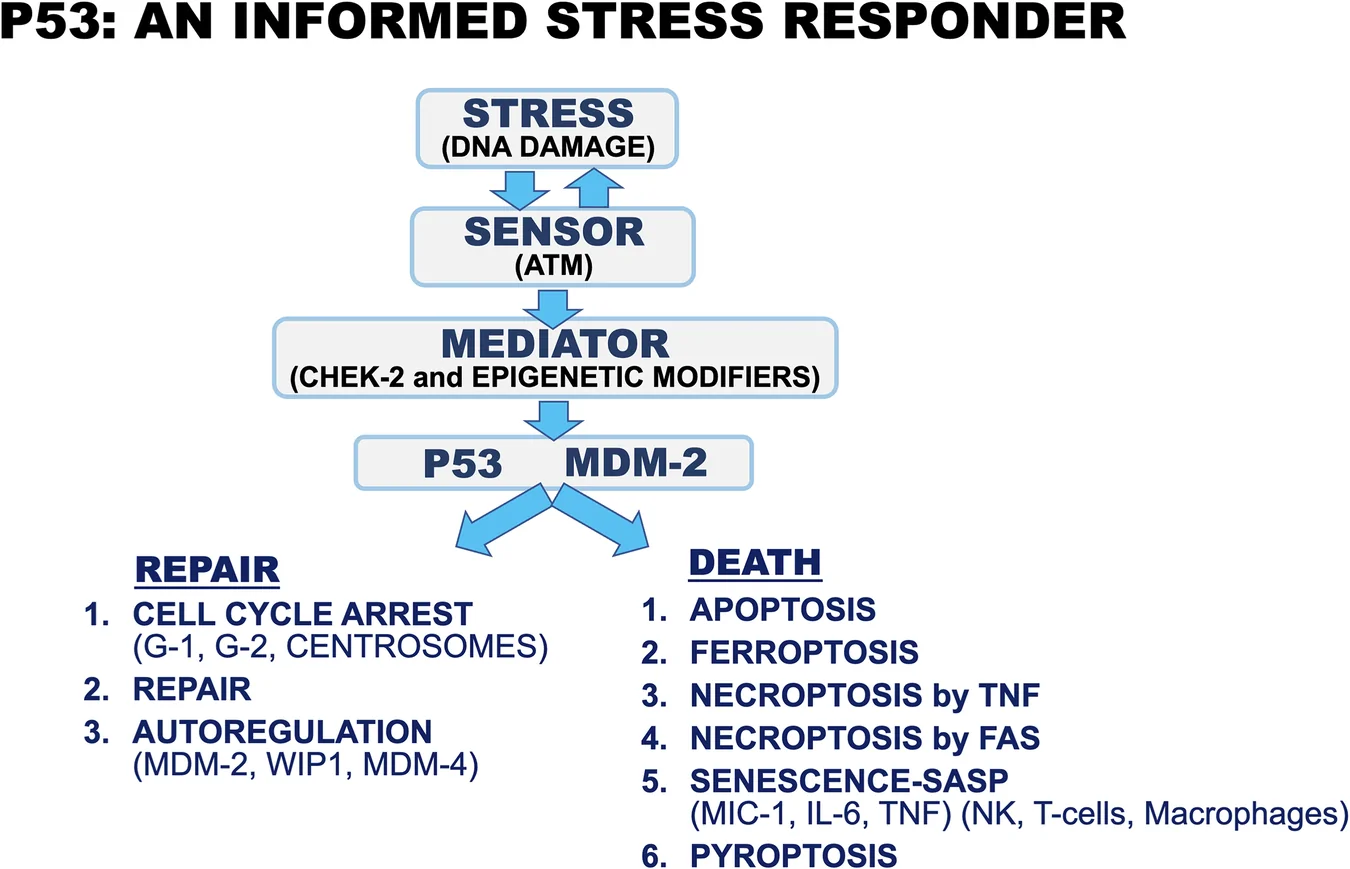
P53: An Informed stress responder. The p53 protein senses single or double stranded breaks in the DNA, with ATM assembling at the break site. An ATM dimer phosphorylates the Chek-2 protein kinase, activating other protein modifying enzymes. Phosphorylation of MDM2 and additional modifications dissociate p53 from MDM2. The short half-life of the p53 protein then increases, resulting in a higher steady state level and increased activity as a transcription factor. Based upon the protein modifications and the p53 splice forms present in the cell, guided in part by the polymorphisms in the *TP53* diversity box, and the dimeric/tetrameric forms of p53 proteins and isoforms, a decision is made to proceed with one of six possible pathways of cell death or cell cycle arrest, DNA repair, and a return to homeostasis for the cell.

The *TP53* gene is a tumor suppressor whose full length p53 protein is a transcription factor. The protein responds to a wide variety of metabolic and genotoxic stresses and arbitrates cell fate decisions, including either killing cells that have genetic alterations or repairing the defects and restoring homeostasis, permitting a faithful replication of cells (Fig. 1). Disabling p53 function in differentiated cells enhances their reprogramming into induced pluripotent stem cells (IPSC) containing persistent DNA damage, resulting in the generation of IPSC at high risk of developing into cancer [5, 6]. A *TP53* mutation in both alleles of a cell permits the removal of cytosine methylation from some CpG dinucleotides in DNA, especially from repetitive sequences such as LINE-1 and ALU repeats. The presence of a wild type *TP53* allele in cells undergoing epigenetic alterations results in cell death by a p53-mediated apoptosis [5]. These mechanisms identify p53 function as a critical barrier to neoplasia and provide a context to the frequent inactivation of *TP53* by mutation or loss of alleles in most forms of cancer.

The key functional characteristic of the p53 protein is its exquisite inducibility in response to a wide variety of stresses, resulting in the transcription of specific, context dependent p53-regulated genes. Upon exposure to various stresses, p53 undergoes multiple posttranslational modifications [7] that enable it to escape proteasome degradation induced by the MDM2 ubiquitin ligase and to accumulate in a transcriptionally active form. After exposure to one or more of such stresses, the p53 transcription factor orchestrates a choice of cell destiny among contrasted fates, either eliminating cells containing potentially oncogenic damage or promoting damage repair and homeostasis [8, 9]. This choice depends upon the transcription of distinct sets of p53 regulated genes involved in different cellular pathways (Fig. 1) [9].

Over 400 p53-regulated genes have been identified, but how p53 operates to make context- dependent choices among this panel is not precisely understood. Conceivably, these contextual decisions involve intrinsic effects caused by qualitative and quantitative changes in p53 itself as well as extrinsic, cooperative effects resulting from the concomitant engagement of other cell-specific pathways.

**Fig. 2:**
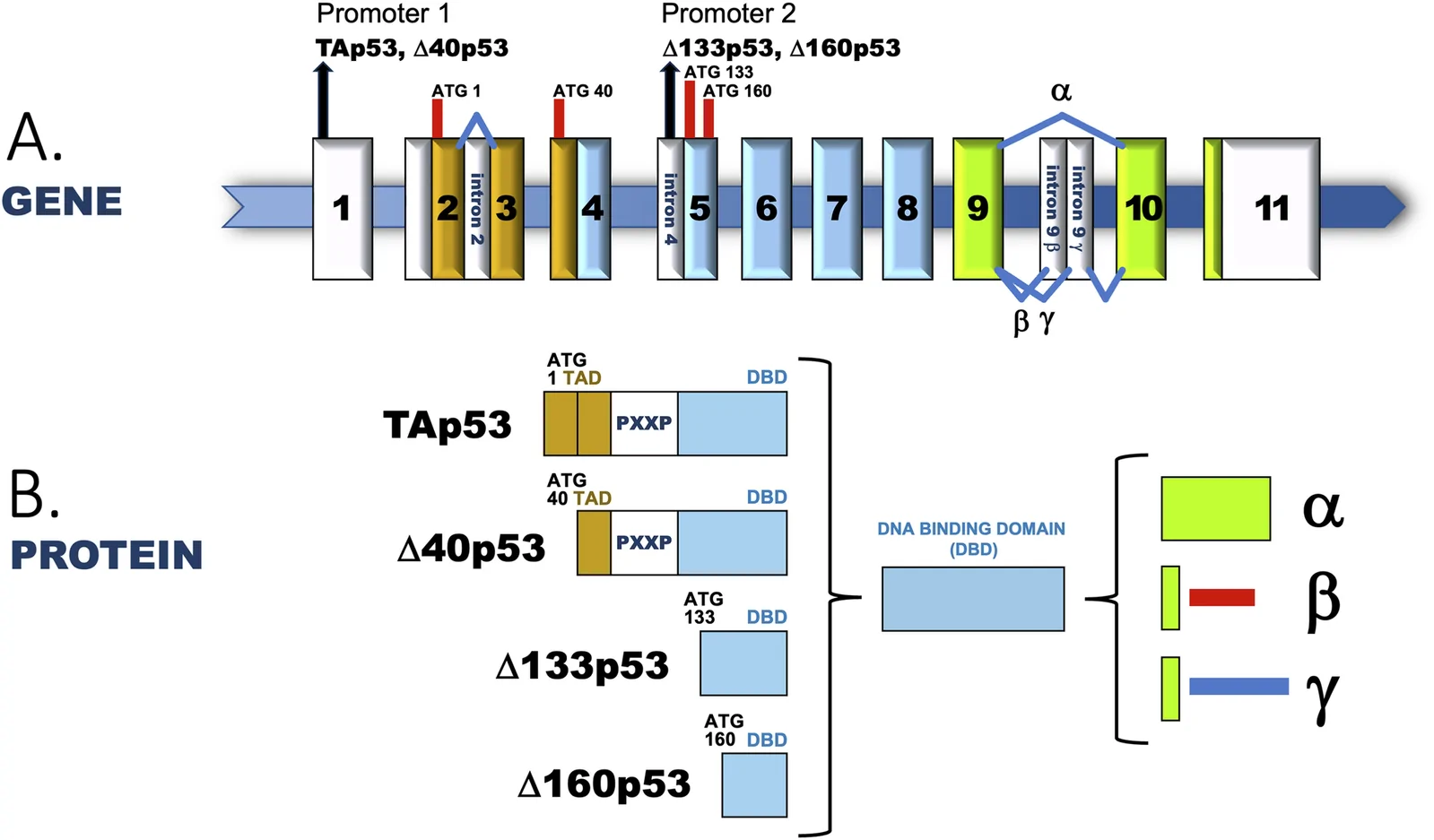
The *TP53* gene and the 12 Protein Isoforms that may be Produced in a Cell. The 20 kb *TP53* gene (not reproduced in scale) with 11 exons in the boxes and introns in the lines between boxes (**A**) and the 12 protein isoforms (**B**) produced from splicing, the full-length m- RNA, the two promoter start sites (P1 and P2), the full-length protein TAP53α and the N- terminal isoforms (∆40p53, ∆133p53, ∆160p53) with their ATG start sites indicated on the gene. The C-terminal isoforms α, β, ϒ are indicated and formed by alternate splicing for β and ϒ in intron 9 and the full length TAP53α having exons 10 and 11 included. The color scheme divides the *TP53* gene and isoforms into the five domains of these proteins; brown is the transactivation domains TAD1 and TAD2, white is the proline domain, blue is the DNA binding domain, green is the tetramerization domain and the regulatory domain. The intron 2 transcript (TP53i2) inserts an additional set of sequences discussed in the text.

Among intrinsic effects, an important source of variability is the fact that the *TP53* gene is potentially expressed as multiple transcripts and protein isoforms [8], caused by a diversity of mechanisms such as the usage of alternative promoters, intron-exon junctions, and transcription or translation start sites. In basal conditions, these complex expression patterns generate low levels of multiple protein isoforms that differ by the length of their N- or C-terminal parts but retain a common segment encompassing most of the DNA binding and the oligomerization domains (residues 160 to 353 out of 393 amino acids) (Fig. 2) [8]. Although the specific physiological functions of each isoform remain largely unclear, some of them, such as the so-called N-delta isoforms that lack variable segments of the N-terminus of p53, appear to antagonize at least some p53 tumor suppressor activities and thus modulate the repertoire of the full length TAp53alpha-dependent responses. By contrast to the tumor suppressive negative functions of the p53 protein, these isoforms may contribute to rescue senescence, repair DNA damage, and provide anabolic functions to promote cell division [10, 11] (Figs. 1 and 2). Other isoforms, such as the beta and gamma isoforms that differ by their C-terminal part, appear to enhance some of the replication effects, in particular by reversing p53-dependent senescence. Thus, it appears that context-dependent cell-fate decisions can be influenced by the coexistence of the canonical p53 protein (TAp53α) with other counteracting or synergistic isoforms (Δ40 or 133β or γ) [8, 10, 11]. Therefore, genetic or epigenetic factors that impinge on the composition and dynamics of the p53 isoform network may have a critical impact not only on cell fate decisions but also on genetic susceptibility to cancer.

This review brings together a large number of diverse observations, made over the past years in the literature, that identify a portion of the *TP53* gene located between exons 2 and 5 of that gene, termed the “p53 diversity box”. This region is composed of about 1200 base pairs, out of a total *TP53* gene of 20,000 base pairs, and harbors a number of gene regulatory signals and polymorphisms which, alone or in combination, may modulate the expression and functions of distinct p53 protein isoforms. In particular, the diversity box harbors 4 exonic protein encoding changes in polymorphisms that specify subtle differences in p53 protein functions, whereas other intronic polymorphisms in this region have been shown to modulate alternative splicing or alternative promoter usage that regulates the expression of p53 isoforms (Fig. 2). Herein we summarize structural, functional, and epidemiological evidence supporting the key roles and mechanisms of this p53 diversity box and we discuss how to test these ideas for a better understanding of the genetic basis of these different polymorphisms and the susceptibilities to develop multiple myelomas in subjects of African and European descent [12].

### P53 protein domains

The human *TP53* gene covers approximately 20 Kb on the short arm of chromosome 17 and includes 11 exons, the first of which is non-coding. These sequences specify a protein product of 393 amino acids, synthesized from a TAp53α m-RNA, whose structure produces a transcription factor, which constitutes the major, canonical form of the p53 protein, identified as isoform TA (transcriptionally active) p53α. It is commonly divided into five domains that are discrete structural and functional units [9].

The first domain (amino acids 1-55, from the N-terminal end) encodes two transcriptional interaction domains, TAD1 and TAD2, which are composed of two distinct sets of residues, or motifs, that bind to the RNA polymerase subunits, the chromatin modifying enzymes, and associated transcriptional regulators. Each of the transcriptional binding signals is composed of several amino acids, but in each case two independent point mutations are required to inactivate each signal: at residues 25 and 26 [13] and at residues 53 and 54 [14]. These two signals control some p53 regulated genes in common and some unique genes at each of the different sites [14].

The second domain of the p53 protein is located between amino acids 55–100 and is called the proline-rich domain [15] because of its high proline content organized in a specific motif. Twelve of the 45 amino acids in this domain are prolines. Four PXXP sequences (P is proline and X is any other amino acid) are found in this domain and a fifth is formed by an Arginine/Proline polymorphism at codon 72. The ARGXXX PXXP sequence and the PXXP signals are protein interaction binding sites, and proteins with SH-3 structures bind to those proline rich sites [15], among which are the GAS1 and FAK proteins, involved respectively in growth suppression and cellular adhesion and mobility [16, 17] bind here. MDM2, the p53 specific ubiquitin ligase, binds to p53 at two sites, one at codons 25, 26 [13] in the first TAD domain and a second site surrounding codon 72, which is polymorphic for proline/arginine [15, 18] in the proline domain. The MDM2 binding to proline at codon 72 is a weak interaction, whereas the binding to arginine is a stronger interaction. Because MDM2 is a ubiquitin ligase that regulates the levels of p53 proteins in the cell, codon 72 proline/proline homozygotes have higher levels of p53 protein in cells than arginine/arginine homozygotes [12, 18]. This results in a number of phenotypes that can have an impact upon cancers in humans.

The third domain is the DNA binding domain, which binds to a set of degenerate DNA sequences in the human genome composed of four palindromes of five nucleotides repeated four times (Pu, Pu, Pu, C/A/T, T/A, G, Py, Py, Py) (-spacer-). This motif is then repeated a second time after a spacer in the genome [19]. A tetrameric p53 protein [20] recognizes two turns of the DNA helix or 20 base pairs composed of four half sites and two palindromes. The unique C and G residues in this sequence are the strongest binding sites in this DNA motif for the p53 protein. These degenerate binding sites permit p53 proteins to regulate the transcription of many genes with different affinities and different times of transcription after activation of the p53 transcription factor, and they produce m-RNAs and proteins at different concentrations. It is estimated that the p53 tetrameric protein might regulate the levels of some 400 or more genes [9, 14].

The fourth domain, called the tetramerization domain, from amino acids 319 to 353, forms a dimer held together by a salt bridge [20] The remainder of the protein (the fifth domain) from amino acids 353 to 393, is called the regulatory domain, which is very basic, having seven lysine residues whose protein modifications (acetylation, methylation) can regulate DNA binding and activity of the transcription factor [10, 21, 22].

### P53 Delta isoforms

There are at least twelve different documented *TP53* mRNA variants and isoforms [8] that selectively produce p53 proteins that are structurally different from TAp53α (Fig. 2). These variants and isoforms can be grouped into N-terminal isoforms (Δ isoforms), initiated at internal AUG codons within the N- terminal section of TAp53α, thus lacking part or all of structural domains 1 and 2, and C-terminal isoforms, resulting from usage of an alternative exon 9, encoded in intron 9, thus having different C- terminal sequences than TAp53α. These isoforms may act either by interacting with and modulating TAp53α activities, or independently, thus expanding the p53 repertoire beyond the genes and pathways directly controlled by TAp53α (Fig. 2). Of particular interest in this respect are the two Δ isoforms, Δ40p53α and Δ133p53α.

Δ40p53α lacks TAD1, the first of the two transcriptional activation domains that also encompasses the first MDM2 binding site. This isoform is produced by two distinct mechanisms, internal initiation at AUG40 from canonically spliced p53 mRNA, or alternative splicing that retains exon 2, leading to the introduction of a stop codon immediately downstream of AUG1 and alternative usage of AUG40. Studies by H. Scrable and her collaborators [23] have shown that Δ40p53α was highly expressed in embryonic stem cells (ESCs) and was the major p53 isoform detected during early stages of embryogenesis in the mouse. In these cells, haploinsufficiency for Δ40p53 caused loss of pluripotency, whereas increased dosage extended progression towards pluripotency and inhibited differentiated states by controlling the activity of full length TAp53α targets such as NANOG and IGF1R1 [23]. On the other hand, the dosage of Δ40p53α appears to be critical for cell senescence and organismal aging. In a particularly interesting set of experiments, Pehar and his colleagues [24–26] have generated transgenic mice in which a genomic fragment corresponding to Δ40p53α is expressed alongside TAp53α. These transgenic mice showed dramatic signs of premature aging, a sharp decrease of lifespan, and a shortened reproductive span [24]. They were also thwarted in their growth and underwent rapid cognitive decline when engineered to express a humanized mouse amyloid precursor protein (APP) [25, 26].

These experiments bring up an apparent contradiction in the literature. The Tp53 knockout mouse, with no TAp53 genes, is viable [27]. Yet the haploinsufficiency of Δ40p53α and the excess levels of Δ40p53α in the presence of a wildtype TAp53α gene have phenotypes that are early age of onset lethality. The explanation for this became clear when it was realized that a molecule of wildtype full length TAp53α formed a dimer and Δ40p53α formed a dimer and they combined to produce a tetramer that produced a different transcriptional phenotype from either a wild-type tetramer of TAp53α or a null-p53 mutant mouse. These delta N-terminal splice forms likely function with full length TAp53α, or even p63 or p73 [28], to produce phenotypes different from full-length p53 tetramers or from a total deletion of the *TP53* gene. It is also possible that some *TP53* gene deletions also express some of these Δ40 or 133p53α or β when these coding sequences remain intact in a genome. They then could enter into mixed tetramers with P63 or p73 [28].

Δ133p53α deletes the first 132 amino acids of the p53 protein eliminating the TAD1 and 2 domains, the proline domain, and that part of the DNA binding domain encompassing the L-1 loop and its two short flanking β-sheets S1 and S2 [29]. By contrast with Δ40p53, which is transcribed from the same pre-mRNA as TAp53α, Δ133p53α is transcribed from a distinct mRNA under an internal DNA promoter (termed P2) located within sequences encompassing intron 2 to exon 5 [29]. This sequence segment contains predicted binding sites for proteins that alter splicing patterns and cell type specificity. The transcription binding site for OCT-1 (ATGCAA, a weaker OCT-1 bind site) is present in exon 4, SOX-9 (AACAAT) is present in exon 4, the half site for the estrogen receptor binding site (GGTCA) spans the end of exon 4 into the start of intron 4 and the binding site for HSF-1 (nGAAnnTTCn) has two sequence matches in intron 2 and intron 4. Each of these transcription factors have been shown to play a role in human cancers, and ER and Sox-9 play gender specific roles. This same region also contains four p53REs (p53 DNA binding responsive elements termed P2, which are degenerate p53 binding sites); RE-1, in the 5’ end of intron 3; RE-2, within exon 4; RE-3 and RE-4, located in intron 4 where these two REs are 9 bp apart and upstream of the initiation start site of transcription through which TAp53α binds and upregulates the transcription of Δ133p53α mRNA. The second of these p53REs in intron 4 also includes an active ER half site that has been shown to regulate a five-fold responsiveness to estrogens [29]. These REs regulate the synthesis of the Δ133p53α protein isoform which can form a mixed tetramer with TAp53α, impairing its capacity to bind and transactivate some, but apparently not all, canonical p53 target genes, thus defining a selective negative autoregulatory loop, much like the negative feedback loop between p53 and MDM-2, its ubiquitin ligase [10, 11, 29]. Importantly, however, mixed Δ133p53α-T53p53α tetramers appear to retain the capacity to bind to DNA and repair DNA damage and carry out telomere repair activity, mitochondrial functions and productivity (the *PGC1*-*α* gene is transcribed adding mitochondrial functions) and metabolic functions (GLUT-3, glucose uptake; SIRT-3, a mitochondrial NADH dependent HDAC are transcribed) [10, 11]. In addition, cells expressing mixed tetramers secrete growth factors that can affect adjacent cells in a non-cell autonomous fashion, inducing senescent cells to regain their ability to divide and function [11]. This has been demonstrated in senescent human T-cells that have failed to kill tumor cells. When Δ133p53α is added to those senescent cells they kill cancerous cells at high target-to-killer T-cell ratios (10 target to 1 killer T-cell) and the cells enhance ribosome biogenesis and promote rapid cell divisions, repairing DNA damage [11]. Thus, the production of this isoform could well be one of the main mechanisms that the p53 protein uses to choose between cell death after a stress, or cellular repair and a return to homeostasis as indicated in Fig. 1 [9, 11].

### The p53 diversity box

**Fig. 3:**
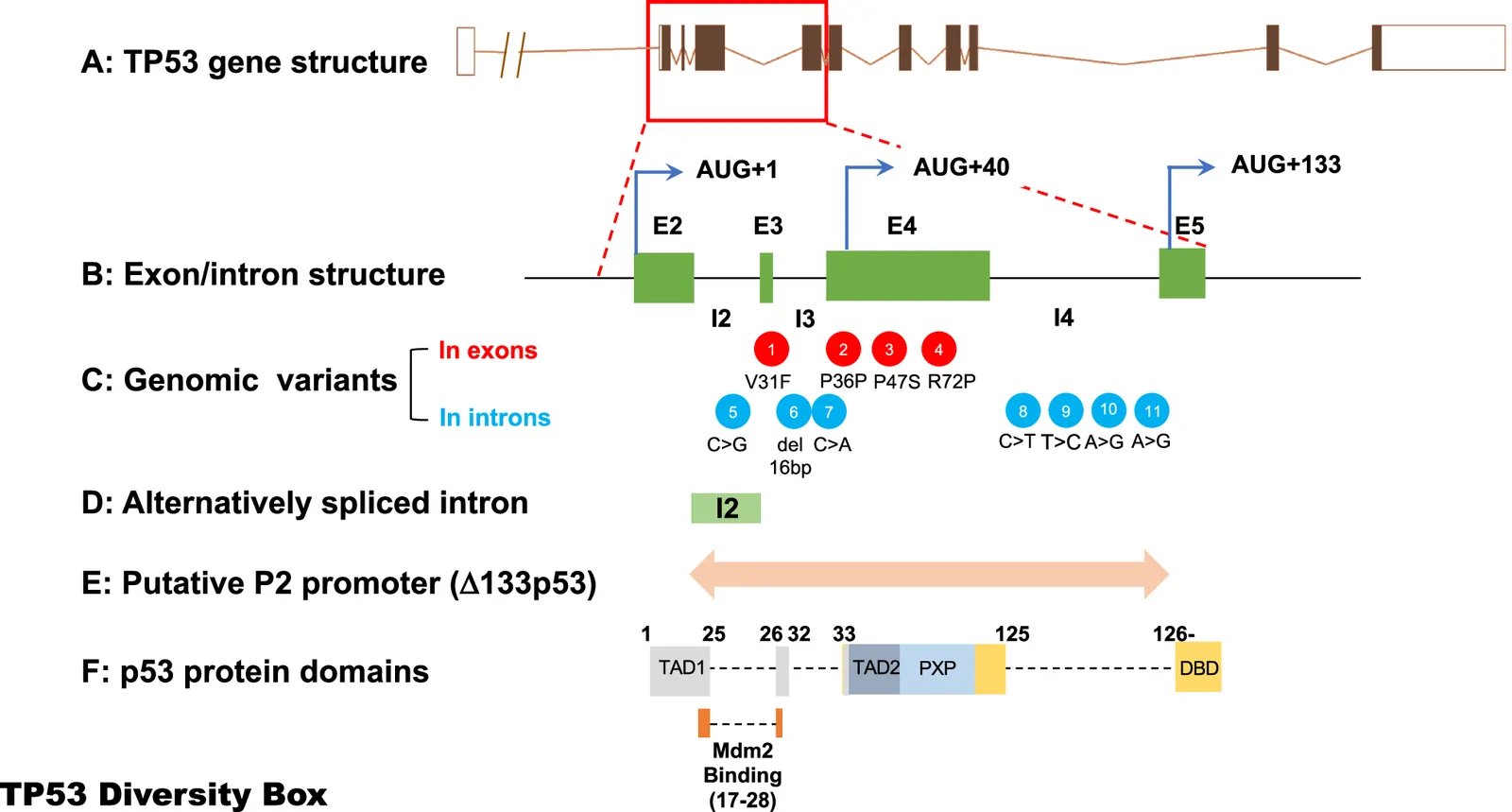
The *TP53* Diversity Box. **A** A representation of the *TP53* Gene with the exons shown as black squares and the intronic region as a line. The square delineates the Diversity Box. **B** The Exon-Intron structure of Exons 2-5. **C** The Polymorphisms discussed in the text with the 4 coding polymorphisms in red and the 7 intronic polymorphisms in blue. **D** The alternatively spliced intron 2 (i2). **E** The pink line shows the extent of the sequences that comprise the P2 promoter which is the start of ∆133p53. **F** The position of the p53 protein domains with one of the MDM2 binding sites to the p53 protein that overlaps TAD-1. The second MDM2 binding site is at codons 72 and 73 in the proline domain and codon 72 is polymorphic for proline/arginine. DBD is the DNA binding domain.

The 1200 bp region of the *TP53* gene that spans initiation codons 1 (AUG) in exon 2 and 133 in the proximal part of exon 5 specifies an impressive combination of genetic, transcriptomic, and proteomic motifs that contribute to the selective activation and cell-fate specificity of p53 suppressor functions (Fig. 3). This region consists of a succession of short introns and exons encoding TAD1 and TAD2, the MDM2 binding sites, the proline-rich region, and the proximal part of the DNA-binding domain. It contains a dense array of posttranslational modification sites (Ser 6, 9, 15, 20, 46, 92, 99, 116; Thr18, 55, 81; Pro82; Lys101) [7] that have been shown to mediate p53 activation or inactivation in response to various forms of stress, and to arbitrate selective cell-fate decisions such as cell-cycle arrest versus apoptosis (Fig. 1 and ref. [8]). It also contains the key regulatory elements that control the selective expression of Δ40p53 and Δ133p53, including alternative AUGs at codons 40 and 133, internal ribosome entry sites (IRES), the alternatively spliced intron 2 that supports Δ40p53 expression, and the internal P2 promoter overlapping and controlling the expression of Δ133p53. Together, these characteristics identify this short DNA segment as a “diversity box” (DB) that includes, interconnects and integrates several molecular mechanisms contributing to p53 proteomic diversity and to the fine tuning of its suppressive repertoire (Fig. 3).

The significance of the DB is underscored by its remarkable genetic diversity. It harbors frequent polymorphisms (mAF >0.1) which are in linkage disequilibrium and show significant variations including the frequent p.R72P variant (rs1042522) and rarer variants V31I (rs201753350) and Y107H (rs368771578) making this region a remarkably high density one of such non-silent SNPs (1/240 bp compared to an estimated average density of 1/10,000–20,000 bp in the remainder of the coding genome). The G allele of rs1042522 (p.R72) is found in 71% of individuals of European (Caucasian) ancestry and in 32% of individuals of African ancestry. The p.P72 variant has been shown to have less affinity for binding to MDM2 than the p.R72 variant and in vitro studies have shown that following DNA damage, the p.P72 variant preferentially promoted cell cycle arrest, senescence and DNA repair, whereas the p.R72 variant more effectively induced apoptosis (see Fig. 1 and Fig. 3 and references 9 and 12). The p.V31I (rs201753350) is exclusively found in eastern Asians (0.1%); its functional significance is unknown. Both p.P47S (rs1800371) [30] and p.Y107H (rs368771578) [31] have altered p53 suppressive properties in vitro and have been qualified as “hypomorph” variants, found exclusively in individuals of African descent. Frequent intronic polymorphisms include:

1. a 16 bp repeat in intron 3 (PIN3, c.96+41_96+56del, rs59758982) that alters a G- quadruplex structure regulating the excision of intron 2 RNA and thus modulating the expression the alternative transcript, p53I2 (p53 intron 2 transcript) [29] that supports the synthesis of Δ40p53, and
2. a G:C variation in intron 2 (PIN2, c.74+38 C > G, rs1642785) in which the presence of C instead of G undermines the stability of the p53I2 transcript [28]. There are several SNPs in intron 4 that alter the sequence of the P2 promoter and have been shown to affect the level of expression of Δ133p53 [28].

Given the known frequency of polymorphisms with phenotypes discussed herein, one might expect that cancers could well harbor spontaneous mutations in the introns of the diversity box (introns 2, 3 and 4). Unfortunately, this has not been explored and reported in standard databases. Perhaps this listing of the high density of important regulatory signals in the introns of the *TP53* diversity box will encourage someone to sequence the introns of the *Tp53* gene from cancers and normal cells.

**Fig. 4:**
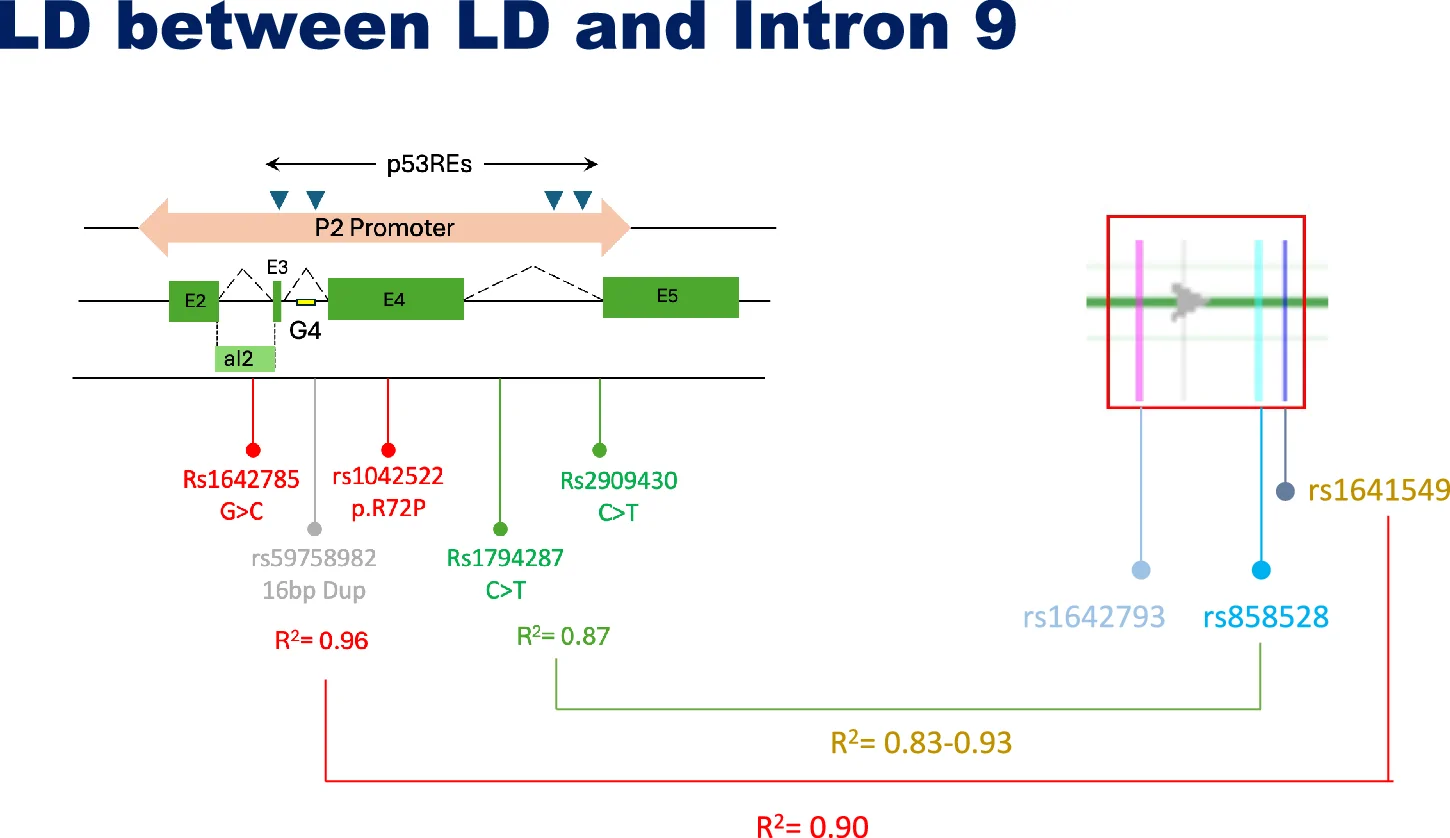
Linkage disequilibrium (LD) between Exons 2 and 5 (The Diversity Box) and Intron 9 of the *TP53* gene. In green are Exons 2, i2, E3, E4 and E5 from the *TP53* gene. Above it in pink is the P2 promotor with the P2 response elements or binding sites of TAp53α producing the m-RNA for ∆133p53.The sequences recognized by the DNA binding domain of TAp53 indicated by the black downward arrows with the entire promoter P53REs (response elements). The P2 promotor is the binding site for the P53 protein to promote the initiation of the ∆133p53α transcript. Below the line are the intronic and exonic polymorphisms in the *TP53* gene indicated by their location in the gene. On the righthand side of the figure, in the red box, are three polymorphisms in intron 9. In red The G/C (Rs1642785) and pR72P (Rs1042522) are in linkage disequilibrium with each other (R^2^ = 0.96) and with rs1641549 in intron 9 (R^2^ = 0.90). The intronic C/T ‘ s (Rs1794287 and Rs2909430 in green) are in linkage disequilibrium with each other (R^2^ = 0.87) and with rs858528 (R^2^ = 0.83-0.93) in intron 9. These polymorphisms travel together, producing protein haplotypes, and insure the correct pairing of the ∆ isoform with the α, β, and ϒ isoforms.

The linkage disequilibrium (LD) among SNPs in the diversity box suggests that this gene segment is under strong selection pressure. In particular, rs1042522 (p.R72P) and rs1642765 (PIN2) are in almost complete LD (r^2^ > 0.9) in three different populations representative of Europeans, Africans, and Asians (Fig. 4). These two SNPs are in partial LD with rs59758982 (PIN3). Likewise, several SNPs in the P2 promoter are in strong LD across these populations (e.g. rs2909430, rs1794287 and rs9895829) (Fig. 4). As a result, the most frequent haplotypes defined by these 6 variants strongly differ in populations of different ancestries, suggesting that in each of these populations different haplotypes have been selected to mediate environmental context-dependent stress responses and cell fate decisions. The African, European, and Asian ancestries have evolved in quite different environments with different selection pressures giving rise to SNPs in the 1,200 base-pair region of the gene [12, 31, 32] The environmental variable with the most impact upon DNA sequences in this region of the proline domain is how direct the sunlight is upon an individual when moving north and south from the equator [32]. The great majority of spontaneous mutations in the proline domain give rise to skin cancers [15]. The sequences of the proline domain differ considerably when diurnal and nocturnal mammals are compared [15]. Skin tone appears to be affected by the proline domain [15]. How different SNPs complement each other and cooperate to confer to each haplotype a specific activity profile is not understood and will require further studies. In any case, these observations support the concept that the DB constitutes a coherent genetic unit that contains integrated signals for adapting p53-mediated cell responses to the specific constraints of different ecosystems [12, 15, 31, 32] Therefore, we propose that genetic variations within the DB may have a strong impact on the differences in genetic susceptibility to cancer that have been observed among groups from different ancestries [12].

### Cell fate decisions arbitrated by the diversity box

How the diversity box operates as a coherent unit is still largely unclear. Given the potential of each of its components to modulate the repertoire of the p53 response, it is tempting to speculate that a critical crossroad in which the diversity box may play a central decision-making role is the question of why the p53 pathway has evolved six different ways to mediate cell death, as seen in Fig. 1. Indeed, beyond their apparently similar outcome (permanently eliminating the target cell), each method for producing cell death has different mechanistic implications and may be adapted to different physiological or pathological contexts. Perhaps each different method of killing a cell interacts differently with the process of degrading the cell corpus and components, resulting in differences in presentation of antigens and outcomes with the immune system. There might also be differences in engaging immune system NK cells, CD4 or CD8 T-cells, inflammatory responses, tolerance, and/or cytokine production, all depending upon the method of killing cells. When a mutant p53 protein in a human triple negative breast cancer with extensive inflammation was treated so as to reactivate the mutant p53 protein to a wild-type p53 protein, the inflammatory response disappeared within a week and the tumor shrank by 41% [33]. Experiments linking the method of cell death by *TP53* to different activations and responses of the immune system are just beginning, and the mechanisms involved in these processes are just being elucidated [34]. For example, p53-mediated pyroptosis has been shown to influence cytokine production (IL-18 and IL-1β), which in turn modulates inflammation and the immune response [35].

Cell- and tissue-specific levels of p53 isoforms may have a strong effect on the transcriptional outcome of p53 activation and on the context-dependent selection of specific suppressive pathways.

In this respect, it is important to keep in mind that TAp53 isoforms are only induced in response to stress. In addition, the Δ40p53 and Δ133p53 lack the protein domain containing the main MDM2 binding site and so are at higher concentrations, and both isoforms can exert dominant-negative effects over TAp53. Therefore, it is conceivable that these isoforms may act as “buffers” to limit, attenuate or modulate the capacity of TAp53 to activate specific components of its transcriptional repertoire. Thus, the characteristics of the DB may affect this decision-making by determining how much active TAp53α will be available, its functional qualities, and the composition of the protein isoform network in which TAp53α operates. These parameters will, in turn, determine the decision- making between different p53-dependent transcriptional outcomes and cell fates. Consistent with this hypothesis, Eiholzer and his colleagues [36] have shown the combination of several SNPs within the DB are associated with cancer risk and with increased levels of Δ133p53β. Specifically, they found that combined heterozygosity at rs1042522 (GC,pR72P) and either of the two intronic SNPs (rs9895829 (TC) and rs2909430(AG)), both located within the P2 promoter, conferred a 2.34–5.35- fold greater risk of developing cancer in a cohort of 689 patients with breast cancer, pancreatic cancer, or glioblastoma. These SNPs were associated with shorter patient survival for glioblastoma and prostate cancer and with tumor-promoting inflammation, as evidenced by high levels of CD163+ infiltrating immune cells. Furthermore, a companion study in 33 normal breast fibroblast cell lines revealed that this combination of SNPs was associated with higher levels of expression of Δ133p53 (which, by inference, was interpreted as Δ133p53β). These observations led Eiholzer and his colleagues [37] to suggest that this SNP combination allows increased expression of the Δ133p53 isoforms to promote the recruitment of immune cells that create an immunosuppressive environment, leading to cancer progression. It will be interesting to determine whether this conducive immune environment may be caused by the choice of different types of p53 suppressive responses, affecting cell degradation and presentation to the immune system. Of note, the specific contribution of rs1042522 (p.R72P) to this effect remains to be elucidated. In addition to its structural effect on p53 protein, this SNP may also affect the conformation of the P2 promoter. However, given that rs1042522 is in almost complete LD with rs1642765 (PIN2), which modulates the expression Δ40p53, it would be interesting to determine whether its effect is not a proxy for a possible involvement of this isoform, alongside Δ133p53. Could it be that these combinations of isoforms expressed in tumors and which have been shown to affect cancer risk and overall survival, also contribute to the mutant p53 “gain of functions” phenotypes? Tumors most commonly have no wild type p53 protein to pair with a p53 mutant N-terminal isoform. But the mutant p53 isomer could form a hetero-tetramer with p63 or p73 [28], and that is a testable idea in these cancer cells [9–11].

The subtle roles of the DB in regulating p53 activation may help to explain the intrinsic differences in p53 levels, stability, and functional responses from one cell/tissue type to another, even in response to stresses of similar nature and intensities. They may also account for the selection of different modalities of *TP53* inactivation as well as different frequencies of *TP53* mutations in different types of cancers. For example, almost 100% of high grade serous ovarian carcinomas harbor *TP53* mutations, suggesting that genetic inactivation (and perhaps induction of a gain-of-function effect) is mandatory for cancer development [9]. By contrast, testicular teratocarcinomas have none or only a small number of *TP53* mutations in the cancer stem cells (embryonal carcinoma cells) [21]. In these cells the trimethylation of three lysine residues in the regulatory domain of the p53 protein, attenuates p53 activity without mutations [22]. In several soft and hard tissue sarcomas, the preferential mechanism of p53 inactivation is amplification and overexpression of the MDM2 gene, observed for example in 80-90% of liposarcomas [9]. This mechanism causes the rapid and selective degradation of the wild-type TAp53 protein.

Somatic mutations can occur in residues within the diversity box and may contribute to altering its regulatory features. For example, codons 72 and 73, 82, 89, and 98 are all somatic hotspot mutations in the proline-rich domain of *TP53* [15]. Their corresponding codons (CCC) are preferential sites of formation of UV-induced cytosine hydrates and pyrimidine dimers, a pro-mutagenic lesion. Thus, not surprisingly, these mutations are detected in skin cancers and are virtually absent in cancers of tissues not exposed to UV. Most of these variations have not been tested for their significance when considering the resulting amino-acid substitutions. However, it is possible that they may also cause alterations in the conformation of the P2 promoter and thus changes in the expression of Δ133p53.

Finally, Δ133p53β may also carry mutations contributing to the gain-of-function effects of mutant p53. Overexpression of Δ133p53 has been reported in a number of diverse cancers, such as breast, pancreas, and several forms of squamous cell carcinomas, often concurrent with *TP53* mutation [36, 37]. Interestingly, whereas the great majority of *TP53* gene mutations in cancers are located in the DNA binding domain, codons 100-132, not coding for the Δ133p53α isoform, occur at a two times lower frequency than codons 133-300, on a per codon basis, suggesting that cancers may preferentially select for mutations that disrupt the DNA binding domain of both TAp53 and Δ133p53, which are the common codons expressed in the full-length molecule and its spliced form.

### Variations in the diversity box and Li-Fraumeni Syndrome

Li-Fraumeni Syndrome (LFS) is an autosomal dominant cancer predisposition caused by pathogenic germline *TP53* variants. Carriers of such variants are at high risk of multiple cancers in infancy and throughout adolescence and adult life. The spectrum of LFS cancers is dominated by brain cancers, soft tissue and hard tissue sarcomas, premenopausal breast cancers, and adrenocortical carcinoma, the latter occurring almost exclusively before 10 years of age. Some evidence suggests that variations in the diversity box may influence the penetrance of the syndrome and, significantly, the age at first cancer onset. In a cohort of French carriers of germline *TP53* variants, Bougeard et al. [38] have shown that the mean age of tumor onset in carriers of two copies of the G allele of rs1042522 (encoding p.R72) and SNP309 in the MDM-2 gene was 21.8 years, compared to 34.4 years in carriers homozygote for the C allele (encoding p.P72) (*p* < 0.005). Given the significance of this modifier effect, there have been surprisingly few attempts at replicating these findings and expanding them to other polymorphisms. Indeed, LFS is a rare disease, with fewer than 2500 carriers and families reported in public databases, making it difficult to assemble unbiased case and control series with sufficient statistical power.

It seems reasonable to assume that the functional consequences of a germline mutation may depend upon the characteristics of the specific haplotype harboring the mutation. On the other hand, carriers of a variant allele can be considered as haplo-insufficient for *TP53*, with the wild-type p53 function contributed mostly, if not exclusively, by their residual wild-type allele, the allele that binds to DNA and transcribes genes. Thus, the capacity of the carrier’s cells to mobilize suppressive p53 functions in response to stress could well depend upon the functional characteristics of the wild-type allele embodied in its haplotype. This hypothesis has been tested in a cohort of Brazilian LFS patients who all carry the same *TP53* germline variant, p.R337H, caused by a founder effect that results in the presence of the same variant allele in thousands of distantly related individuals [38]. Cancer phenotypes within this cohort are extremely heterogeneous, with incomplete penetrance and many individuals presenting partial LFS traits, consistent with the idea that the wild type allele contains polymorphisms that determine these phenotypes [39].

## Conclusions

Although we do not know how many different *TP53* haplotypes there are in the many diverse populations with ancestries from Africa, Europe, Asia, and South America, we do know that these diverse haplotypes may well give rise to several different phenotypes in those populations. These haplotypes in the diversity box have properties which: 1. regulate the levels of wild type full length p53 proteins depending upon the cell type they reside in, and 2. regulate the splicing patterns observed in the cell types they reside in. In turn, these two variables can have an impact upon the rates of cell division and the frequencies of spontaneous mutation in the *TP53* DNA binding domain as well as selection for mutant clones in a population. Different haplotypes may well have different mutation rates in the *TP53* gene because the wild type p53 protein levels will differ, and a population of cells with a high level of tumor suppressor activity would select for a rare replicating cell with better efficiency. This could be the explanation for why the mutation frequencies of the *TP53* gene differ (sometimes higher, sometimes lower, depending upon the tissue) in identical tissue-type cancers of African and European ancestries [12]. Different haplotypes can influence the predisposition of cancers in a population [36–39]. For example, in equal population sizes, more than twice as many individuals of African ancestry will develop multiple myelomas over a lifetime as individuals of European ancestry [12]. The p53 protein plays an important role in tissues that undergo recombination, such as B-cells, T-cells, and the germ line, normally by being activated by DNA double stranded breaks, but in the cases of these developmental pathways, p53 must be turned off to give rise to viable differentiated cell types. If the p53 protein was active, it would kill cells with a double strand break in its’ DNA. The efficiency of that process could differ for individuals from diverse ancestries.

Can we quantitate how much information the *TP53* gene contributes to the organism? 1. This information comes from the full-length sequence of the p53 protein (393 amino acids). 2. There are twelve different spliced forms of *TP53* gene m-RNA that produce twelve protein isoforms (Fig. 2) [8].

The diversity box contains four polymorphisms that affect the coding region of the p53 protein and seven polymorphisms in the introns of the *TP53* gene the phenotypes of which we have elucidated in this manuscript (Fig. 3). There are three additional polymorphisms in intron nine that interact through linkage disequilibrium with several polymorphisms in the diversity box and appear to regulate which C-terminal end of the p53 protein that will be employed in the isoforms produced (Fig. 4). These polymorphisms influence the levels of full-length p53 m-RNA and protein to be produced; they influence which splice form is produced to give rise to an isoform. 4. Some of these different isoforms and the full length p53 protein can form monomers, dimers, and tetramers. In turn, the dimers of full-length p53 and the dimers of various spliced forms produce isomers that can join to form mixed tetramers that have their own transcriptional profiles different from the full-length p53 tetramer. [9–11]. 5. Finally, in addition to Δ40, or Δ133 isomers producing heterodimers with full length p53 protein dimers, we know it is possible that the N-delta isomers could form dimers that interact with p63 or p73 dimers (with P63β, ΔNp63α and β, ΔNp73γ) [28], possibly producing yet another set of transcriptional activities. There is very little evidence demonstrating that this occurs in cancers and no evidence yet for possible novel transcriptional profiles. If this should occur we will need to explore new cell types: p63 is at high levels in squamous epithelial cells, and p73 is active in cells that form flagella (lung, germ line and in the nervous system).

**Fig. 5:**
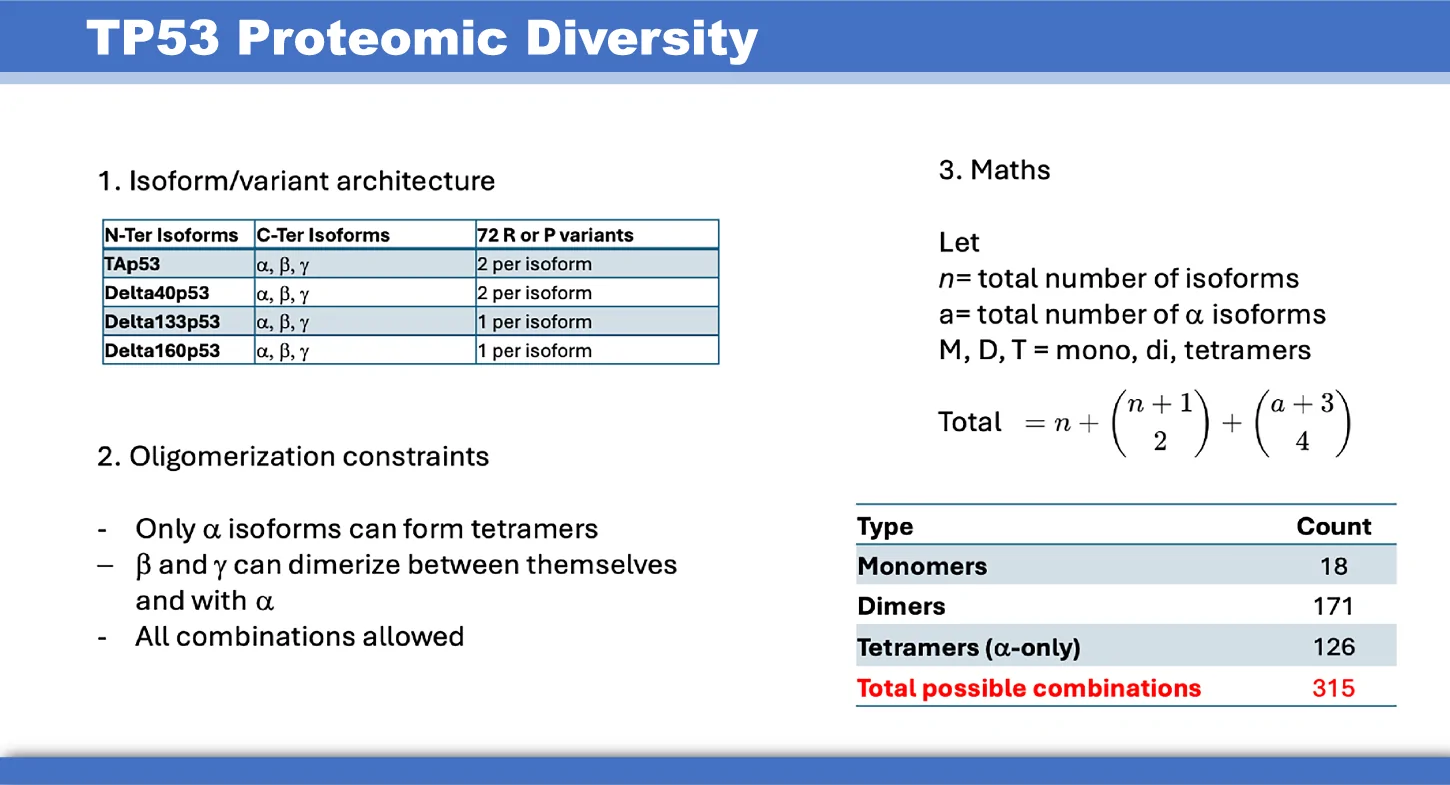
Calculation of the number of unique p53 functional units. The *TP53* gene encodes four N-terminal isoforms (TAp53, ∆40p53, ∆133p53, and ∆160p53) and each of these have three C-terminal isoforms (α, β, ϒ), giving 4×3 = 12 protein isoforms due to alternate splicing of the mRNA. Additionally, the TAp53 and ∆40p53 isotypes come in two variant forms (P72 and R72), providing an additional six isotypes. Therefore, there are a total of 12 + 6 = 18 unique isoforms. The p53 protein may form monomeric (one protein), dimeric (two proteins), and tetrameric (four proteins) functional units. Although all 18 isoforms can form monomers and dimers, only the four α forms can form tetramers. Given these data, we can calculate the number of unique p53 functional units as the sum of all possible monomer, dimer, and tetramer combinations with replacement of the N-TER isoforms with alpha, beta and gamma C-TER, since isoforms can self-assemble. This calculation produces a total of 315 unique p53 functional units.

This manuscript has explored this information using the literature describing the *TP53* gene. We can now ask the question how many different combinations exist to give rise to possible different phenotypes or outcomes? Fig. 5 shows the mathematical calculations for combining each contribution listed above to the total amount of information contained in this gene. The conclusion is that there are at least 315 different combinations of sequences and protein complexes formed from different isotypes. This does not include the pairing of the p53 isoforms with p63 or p73 because clear evidence of novel transcripts in normal or cancer cells is still lacking. It also does not take into account the imposition of linkage disequilibrium, which reduces the possible number of combinatorial polymorphisms in a population (Fig. 4). There are clearly many other proteins from diverse signal transduction pathways that TAp53 and its isomers interact with, so this number (315) should be considered the minimum starting point for that calculation.

There is yet another way in which the p53 protein may be regulated in a cell. The activated p53 protein transcribes the MDM2 gene, which then produces a specific ubiquitin ligase modifying the p53 protein for rapid degradation [9, 13]. This type of circuitry produces an oscillation in the concentration of the p53 protein that is 180 degrees out of phase with the oscillation of the MDM2 protein. A high steady state level of the p53 protein occurs when the p53 and/or MDM2 protein(s) are modified by phosphorylation, MDM-2 no longer binds to p53, and no longer ubiquitinates the p53 protein, so p53 proteins are not degraded. The transcriptional pattern observed when the *TP53* protein oscillates gives rise to DNA repair, whereas a high steady state level of the p53 protein gives rise to senescence and/or cell death [40, 41]. This is likely connected to or reinforced by the codon 72 arginine/proline SNP, which has also been shown to have the same phenotypes and outcomes [9, 12, 15, 16].

We do not know the history of the evolution of these polymorphisms in the *TP53* gene, when they occurred, in what populations, or where those populations resided. By employing populations of African, European, Asian, and Hispanic ancestries that are alive today, there appear to be 5-6 major *TP53* haplotypes that are in the majority of human populations, but there is a good deal of variation in each of those populations, and there are novel haplotypes in some inbred subpopulations. Large, focused DNA sequencing genome projects will begin to sort out some of these questions. But it is remarkable how much potential information is stored in the *TP53* gene that permits the gene to take advantage of the changes in the ecology as humans move about and change their environments.
